# Spatial Representation of Ordinal Information

**DOI:** 10.3389/fpsyg.2016.00505

**Published:** 2016-04-07

**Authors:** Meng Zhang, Xuefei Gao, Baichen Li, Shuyuan Yu, Tianwei Gong, Ting Jiang, Qingfen Hu, Yinghe Chen

**Affiliations:** ^1^School of Psychology, Beijing Normal UniversityBeijing, China; ^2^Institute of Developmental Psychology, Beijing Normal UniversityBeijing, China; ^3^Max Planck Institute for PsycholinguisticsNijmegen, Netherlands; ^4^Beijing Key Laboratory of Applied Experimental Psychology, School of Psychology, Beijing Normal UniversityBeijing, China

**Keywords:** SNARC effect, spatial representation, ordinal sequences, Chinese color words, numerical cognition

## Abstract

Right hand responds faster than left hand when shown larger numbers and vice-versa when shown smaller numbers (the SNARC effect). Accumulating evidence suggests that the SNARC effect may not be exclusive for numbers and can be extended to other ordinal sequences (e.g., months or letters in the alphabet) as well. In this study, we tested the SNARC effect with a non-numerically ordered sequence: the Chinese notations for the color spectrum (Red, Orange, Yellow, Green, Blue, Indigo, and Violet). Chinese color word sequence reserves relatively weak ordinal information, because each element color in the sequence normally appears in non-sequential contexts, making it ideal to test the spatial organization of sequential information that was stored in the long-term memory. This study found a reliable SNARC-like effect for Chinese color words (deciding whether the presented color word was before or after the reference color word “green”), suggesting that, without access to any quantitative information or exposure to any previous training, ordinal representation can still activate a sense of space. The results support that weak ordinal information without quantitative magnitude encoded in the long-term memory can activate spatial representation in a comparison task.

## Introduction

One of the hottest recent debates in cognitive psychology is whether or not symbolic processing, such as the representation of numbers and concepts, causes mental/bodily simulation of perceptual features or properties (e.g., space or location) of linguistic symbols beyond mere semantic processing (Barsalou, 1999). Two decades ago, Dehaene et al. (1993) found that manual responses to larger numbers were faster with the right hand than with the left one, while responses to small numbers showed the opposite. The Spatial-Numerical Association of Response Codes (SNARC; Dehaene et al., 1993) effect reveals that symbols such as numerals are spatially encoded and that magnitude interacts with representational space (Walsh, 2003). This left-to-right representation of small vs. large numerical magnitudes was both internally distributed in the mental number line (Restle, 1970) as well as externally distributed in the physical environment, as most cultures that share left-to-right reading/writing direction also favor the small-to-large number alignment in that direction.

There has been some evidence that the spatial component of number representation can be extended to tasks that do not explicitly require processing of numerical information *per se*. Besides the classic parity judgment task (Dehaene et al., 1993) and magnitude comparison task (Fias et al., 1996), SNARC-like effects can be elicited when digits serve merely as a background distracter (Fias et al., 2001) and the presentation of a mere single digit as a prime can cause spatial shifts of selective attention (Fischer et al., 2003). There was also evidence that manipulated lateral head turning affects random number statement generation as in people turning their heads to the left state smaller numbers than those who turn to the right (Loetscher et al., 2008). In reverse, seeing smaller or larger numbers can influence random lateral movement, such as smaller numbers stimulating leftward movement and larger numbers causing opposite stimulation (Vicario, 2012).

Beyond demonstrating a reliable SNARC effect during explicit and implicit numerical processing, however, there have been some controversies regarding whether spatial-numerical associations are exclusively connected with numbers or whether they can be produced by non-numerical stimuli that are sequentially ordered (Dehaene et al., 1993; Fias and Fischer, 2004; Gevers et al., 2004). At the very least, numbers can convey not only quantity (e.g., eight athletes), but also ordinal information (e.g., the eighth athlete) or even nominative information (e.g., athlete number 8). Earlier findings (Dehaene et al., 1993) suggested that there was no reliable SNARC effect for letters when participants had to classify letters as vowels or consonants, suggesting ordinal information without explicit reference to numerical quantities was not spatially represented. However, when instructing participants to judge whether letters (E, G, I, L, R, U, W, and Y) from the alphabet were before or after a reference letter O, Gevers et al. (2003) found a reliable SNARC-like effect for letters. Also, they revealed that there was robust evidence for the SNARC effect for ordinal information for “months of the year” when participants were required to judge whether a month was before or after the reference month *July*. In this stream of research, number sequences, months of the year and the alphabet are recognized in almost all cultures and are spatially organized in long-term memory. They require minimal training in experimental tasks to elicit the SNARC effect.

Other lines of research show that arbitrarily organized orderly sequences were not readily stored in the long-term memory but were instead learned by training, in order to trigger a spatial representation temporarily available within the working memory (the working memory account, van Dijck and Fias, 2011). For instance, van Dijck and Fias (2011) instructed participants to memorize a randomly ordered number vs. fruit and vegetable word sequence in their working memories before engaging in a parity judgment task vs. a fruit–vegetable judgment task. For both tasks, the results revealed SNARC effects, suggesting that the ordinal information for numeric and non-numeric sequences, temporarily stored in the working memory, was also coded spatially (see also Lindemann et al., 2008). Moreover, Previtali et al. (2010) found an association between ordinal position of the trained arbitrary items and spatial response preference (i.e., the SNARC effect) in order-relevant tasks. In comparison with numbers, whose spatial representations were automatically retrieved from the long-term memory, numerical-spatial associative strength for non-numerical stimuli or arbitrary sequences was weaker, but it could be robust in working memory. The results above indicated that spatial representation of orderly information *can be* temporarily activated in the working memory given an appropriate task (i.e., order-relevant comparison/classification).

However, several question are still unanswered by previous studies. Firstly, it could still be argued that elements, such as months of the year or letters (Gevers et al., 2003), might still contain certain amounts of quantitative information besides ordinal information. During the task, *December* could be translated into the *number twelve* while being interpreted as *the twelfth month of the year*. In addition, letters near the beginning of the alphabet could be coded numerically. For instance, the letter E could possibly be perceived as “the fifth letter,” whereas letters in the middle or the end of the alphabet are less probable to be coded numerically. Nevertheless, the exact position in the alphabet is not always easily accessible consciously. It remains unclear whether ordinal sequencing without explicit or implicit one-to-one mapping of numbers *can* indeed activate corresponding spatial representation, as demonstrated in the SNARC effect. Secondly, while pre-task training to memorize a sequence can be helpful for activating ordinal representation in the working memory, this study aimed to investigate ordinal representation without pre-task training that might have introduced extra memory load.

To test the spatial representation of ordinal information that is uncontaminated by quantitative information and that might impose minimal memory demand on the judgment task, one novel type of non-numerical orderly stimuli – Chinese color word sequence – was adopted in the present study. In China, the color spectrum, “red, orange, yellow, green, blue, indigo, and violet” and in Chinese as “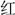, 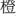, 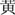, 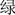, 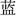, 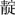, 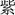
” is first introduced either in art class as alluding to the palette or in science classes with reference to the rainbow in primary school curriculums. It was then taught again in high-school physics and chemistry classes in the context of the optical spectrum of matter. It is worth noting that although this knowledge has been instructed sequentially, neither spatial nor ordinal information of these color words was explicitly mentioned or emphasized. Similar to the acronym ROY-G-BIV (**R**ed, **O**range, **Y**ellow, **G**reen, **B**lue, **I**ndigo, and **V**iolet) as a memory cue for the color spectrum in the English language, the sequential organization of the color words in Chinese was nothing more than a mnemonic technique to facilitate the verbatim retention of the scientific facts. This sequence is not arbitrary or random, and yet contains less distinctive ordinal information compared to months and letters (red is very improbable to be memorized as “the first color”), as it was less frequently used and was created as part of the culturally specific idiosyncratic pedagogy. Importantly, compared with months and letters, the Chinese color word sequence also does not have direct reference to numeric magnitude. In fact, most Chinese are so familiar with the sequence that they can retrieve it effortlessly without intentional recollecting, making direct retrieval of magnitude information even less likely, if not completely impossible.

Last but not least, most Chinese characters are polysemous (i.e., having multiple meanings). Chinese color words are no exceptions and each color word can have its literal color referent or refer to something metaphorically, sharing certain features of that color. For instance, red (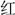) and violet (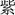) can both appear in the four-character idiom “
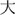

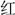

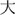

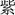
 (meaning “very red and very violet” literally)” at the same time, meaning “very popular,” because both colors are considered conspicuous in the culture. In sum, because of schematic storage in the long-term memory and polysemy richness in Chinese, Chinese color sequence is ideal for testing the spatial activation of ordinal information apart from direct spatial-numerical association during order-relevant tasks.

It was hypothesized that with proper task demand (i.e., order comparison), a SNARC effect would exist for a sequence with weak but non-arbitrary ordinal information (as compared to numbers, months, or random word sequences) in the long-term memory. This was consistent with a universality perspective regarding the SNARC effect that ordinal information is also spatially represented (Gevers et al., 2003, 2004) that is not selective or specific to numerals or quantities alone (Dehaene et al., 1993). Referring to Gevers et al. (2003), the distance between the target and the reference, the side/hand of response and position in relation to reference (before vs. after) were manipulated in an order comparison task to investigate the SNARC effect for concepts conveying uncontaminated ordinal information.

## Materials and Methods

### Participants

Twenty-six right-handed Chinese-speaking students at Beijing Normal University (14 males) participated in the experiment after signing an informed consent form. The average age was 20.77 years with an SD of 1.88 years. The participants were recruited through an online advertisement and they had normal or corrected vision. Each participant received 10 RMB as a reward after the experiment. The experiment protocol was approved by the IRB of Beijing Normal University.

### Stimuli and Design

**FIGURE 1:**
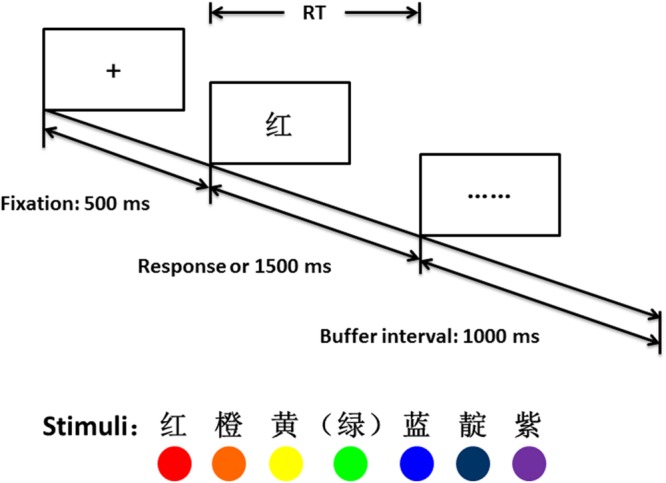
**The procedure of one trial in the experiment.** Corresponding color words and colors are shown at the bottom.

Six Chinese color words (*red, orange, yellow, blue, indigo*, and *violet*) were used as targets (see also **Figure 1** for materials) with *green* as the reference color word in the comparison task (i.e., *before* or *after* green). Three within-subject independent variables were (1) Position (before or after reference), (2) Side of response (left or right hand) and (3) Distance from reference [1, 2, and 3 unit(s)]. Red (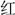) and violet (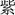) were assigned to distance [3] because they are 3 units away from the reference “green,” and similarly, orange (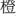) and indigo (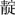) to distance [2] and yellow (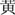) and blue (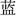) to distance [1]. Two blocks of different responding keys were manipulated. In one block, participants pressed the left key for “before-green” targets and the right key for “after-green” targets, and, in another block, the opposite fashion. The order of blocks were counterbalanced across participants.

### Procedure

The experiment processed with SuperLab 4.0 on a Sony VAIO laptop in a quiet lab room. Subjects were seated approximately 50 cm from the screen and participated in two blocks of order judgment task. They were asked to judge whether the position of each target stimulus presented was before or after “
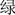
(green)” in the Chinese color word sequence by pressing two buttons on the keyboard (“e” for the left hand and “p” for the right hand) with their index fingers. Both speed and accuracy were emphasized. During the task participants were tested twice: once with colors before green assigned to the left hand key (compatible block) and once with colors before green assigned to the right hand (incompatible block). The order of the two blocks were counterbalanced across subjects.

In each trial, a ‘+’ (width 2.0°, height 2.0°) was presented as a fixation mark for 500 ms (see **Figure 1** for procedure), immediately followed by the target color word (width 4.2°, height 4.2°), until a response was given or 1500 ms elapsed. The screen then was replaced by ‘……’ as a buffer interval for 1000 ms, after which a new trial would start. Each of the six color words was presented eight times in random order for each block. The whole experiment took approximately 8 min to complete.

## Results

**FIGURE 2:**
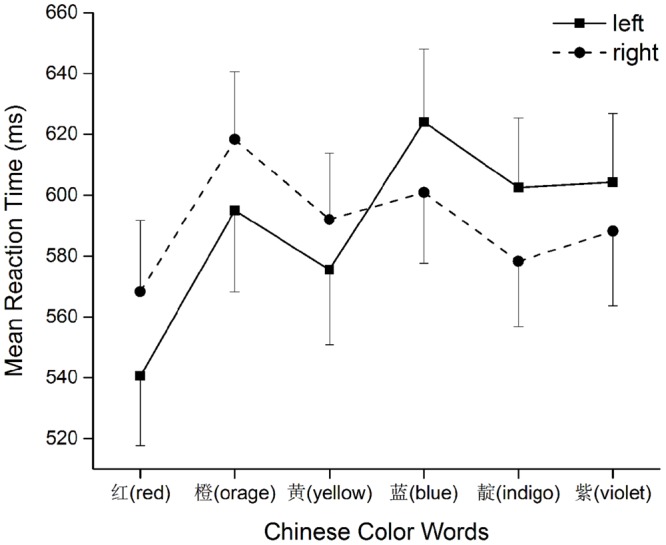
**Response times for left and right hand of each color**.

Trials with no response or with reaction time less than 200 ms (ref. Gevers et al., 2006) were deleted (0.96%). Mean error rates were 4.6, 6.5, 6.0, 7.2, 5.0, and 4.8% for red, orange, yellow, blue, indigo, and violet, respectively. Error response trials were then eliminated from formal data analysis (5.7%). In addition, RTs that were more than 3 SD were excluded (2.0%). Mean correct RTs were 557, 611, 591, 618, 593, and 601 ms for each color in order (see **Figure 2**).

A three-way ANOVA with Position (before or after reference), Distance from the reference color “Green” (1 for yellow and blue; 2 for orange and indigo; and 3 for red and violet) and Side of response (left or right) was conducted. Firstly, there was a main effect of Position, *F*(1,25) = 7.83, *p* < 0.05, η^2^ = 0.24; a main effect of Distance, *F*(2,50) = 10.43, *p* < 0.001, η^2^ = 0.29. There was an interaction between Position and Distance, *F*(2,50) = 10.97, *p* < 0.001, η^2^ = 0.30. Simple effect analyses showed the effect of Distance had different patterns on each side of green. Before the reference, red was responded to faster than orange and yellow, Bonferroni *p*s < 0.01; after the reference, only indigo was significantly faster than blue, Bonferroni *p* < 0.05. In addition, red was responded to the fastest among all color words, *p*s < 0.01, due to its position at the beginning and more frequent use in Chinese than other color words. Other comparisons did not reach a significant standard, *p*s > 0.05. On both sides of the reference, the pattern of RT indicated an effect of distance though not all pairs of comparison were significant. Nevertheless, the relatively slower response to color words close to the reference green and faster to those far from it suggested that participants solved the task by comparing each color word to the reference (see Moyer and Landauer, 1967; Gevers et al., 2003).

Most importantly the Position × Side of response interaction was significant, *F*(1,25) = 8.42, *p* < 0.01, η^2^ = 0.25, indicated an order-spatial association for non-numerical stimuli (**Figure 2**). Simple effect analyses demonstrated significant opposite effects of Side of response at before-reference positions, *F*(1,25) = 4.39, *p* < 0.05 and at after-reference positions, *F*(1,25) = 6.60, *p* < 0.05, reflecting that left-hand responses were faster than right-hand ones to targets that were before reference position and vice versa.

**FIGURE 3:**
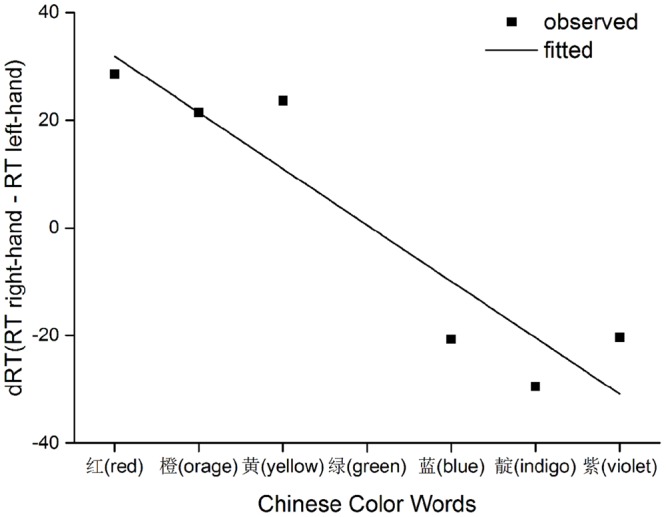
**Observed data and fitted lines representing RT differences between two hands as a function of sequential position**.

Lastly, we performed a regression analysis to give a direct look at the SNARC-like effect (Fias et al., 1996) and then another ANOVA to quantify its linear effect size (Pinhas et al., 2012; Tzelgov et al., 2013). In order to catch the essence of SNARC effect as a function of the order of color words (i.e., 1–7, except 4 for green), a separate regression of mean dRT (right-hand response minus left) for each participant onto color orders was conducted (Fias et al., 1996; Gevers et al., 2003, 2004). This resulted in the following equation as plotted in **Figure 3**: dRT = 42.35–10.46 × (Order). The regression reached significance with *F* = 25.57, *p* < 0.01, adjusted *R*^2^ = 0.93, and with Order contributing significantly, *t*(25) = -5.06 and *p* < 0.01, to the dRT variances. Moreover, as suggested by Pinhas et al. (2012) and Tzelgov et al. (2013), the linear trend of SNARC effect was significant, *F*(1,25) = 9.72, *p* < 0.01, with the linear effect size equals 0.28. In sum, converging evidence supported that the left-to-right spatial representation of rather abstract non-numerical concepts were activated in a task requiring direct evaluation of relative orderly information.

## Discussion

The purpose of the study was to examine whether Chinese color words were able to activate the spatial coding of orderly information in the working memory, as were those heavily trained or temporarily memorized (i.e., numbers, months, days of the week, and arbitrary sequence, Dehaene et al., 1993; Gevers et al., 2003, 2004; Van Opstal et al., 2009; Previtali et al., 2010; Huber et al., 2016). Our results revealed that color words from the beginning of the sequence were responded to faster with the left hand than with the right hand, whereas the reverse pattern was obtained for color words toward the end of the sequence. The finding suggested that encoding of familiar ordinal concepts was spatially stored in the long-term memory and could become activated in the working memory during an appropriate task, just as in a training paradigm.

In line with the findings of Gevers et al. (2003), the SNARC-like effect we obtained indicated that like numbers and letters, the Chinese color word sequence could be spatially coded for certain task demands. This was quite intriguing especially considering that Chinese color words could convey meanings besides color alone, and therefore its ordinal information was weaker compared to months of the year, days of the week or even letters in the alphabet. Color words (in both English and Chinese) were not on ratio scales as numbers or interval scales as months or days.

We observed a main effect of distance in the analysis of RT for color words. Though the distance effect was not typical (not all comparisons between color word pairs were statistically significant), the pattern of RT data did demonstrate a trend that elements close to the reference color word were responded to slower than those far from the reference. The relationship between dRT and position of each color word (illustrated in **Figure 3**) also seemed like two horizontal lines differing by intercept, which resembled the findings of Gevers et al. (2006). Gevers et al. (2006) mentioned a difference between data patterns detected from parity judgment task and magnitude comparison task on numbers. In parity judgment task, the relationship between dRT and number magnitude was a linear one, whereas in magnitude comparison task, the relationship showed a categorical pattern. Gevers et al. (2006) provided an explanation that the categorical shape was the result of an interaction between the distance effect (in the magnitude comparison task) and the time course of the SNARC effect. Similarly, the current study also employed a comparison task (of sequential order). Our data pattern of relatively slower RT for elements close to green and faster for elements far from green might have been the cause of the categorical shape.

It might be argued that color can be defined as the wave length or frequency on the optical spectrum. It is true that the order of each color is defined by descending order of wave length or ascending order of frequency. However, one could not tell measurably how far red was from orange in the sequence either mentally or physically but be aware that red is somehow arranged in front of orange. Chinese color words are notations arbitrary for the basic visible colors but not for precise ranges of wave lengths or frequencies, so the possibility is rather low that wave length or frequency information could be directly activated with representation of color words. This study would even suggest a SNARC-like effect with an opposite orientation against the current result with wave length activated numerically.

Because of its culture-specific and non-arbitrary nature, the Chinese color word sequence was suitable for testing spatial-positional association without training. Previous studies demonstrated that temporarily established number sequences with non-default order could elicit spatial-positional association and sometimes eliminate the spatial-numerical association (Lindemann et al., 2008; van Dijck and Fias, 2011). A recent study have demonstrated that these two kinds of associations coexisted when participants were asked to memorize temporary number sequences (Lindemann et al., 2008; Huber et al., 2016). Nevertheless, the working memory paradigm demanded a large amount of training and cognitive load on participants and the experiments normally took an excessively long time (e.g., Huber et al., 2016). In comparison, Chinese color words was a suitable sequence to test the spatial representation of ordinal information in Chinese subjects because they were familiar with the Chinese color word sequence already and it required no extra attentional effort of memorization to follow the task demand. Although the activation of the spatial representation of Chinese color words was more spontaneous than that detected through training paradigms (Lindemann et al., 2008; van Dijck and Fias, 2011), the current study did not address the levels of automaticity of color word spatial representation, which may require another study using an order-irrelevant task.

Finally, it is not the intention of this paper to explore the boundary conditions (e.g., order comparison vs. vowel/consonant classification task) under which the SNARC effect may or may not occur for ordinal sequences (for a review, see Cohen Kadosh et al., 2008), which deserves another full research article in its own right. After all, the degree of automaticity of the spatial representation of varying stimuli by manipulating task demand differs from whether the SNARC effect is selective to numerals and are thus quantity-specific or reflect more universal senses of spatial organization for any given pre-ordered information. Therefore, the present finding that non-numerical, non-random ordinal information *can* still activate spatial representation with the appropriate task demand was nevertheless non-trivial, as it suggested that the task-modulated SNARC effect at least partially, if not in whole, reflects the spatial distribution of any ordinal sequence, which might not be number-specific or even magnitude-specific (Dehaene et al., 1993; van Dijck and Fias, 2011). In contrast, consistent with Gevers et al. (2003, 2004), this study further demonstrated without any potential confounds that semantic/conceptual processing of quantitative information or temporarily learned order is not the prerequisite for generating the spatial representation of ordinal information.

## Author Contributions

MZ developed the study concept. All authors contributed to the study design. Testing and data collection were performed by MZ and TJ. MZ, BL, SY, TG, and XG performed the data analysis and interpretation under the supervision of TJ, QH, and YC. MZ drafted the paper, and TJ, TG, and QH provided critical revisions. All authors approved the final version of the paper for submission.

## Conflict of Interest Statement

The authors declare that the research was conducted in the absence of any commercial or financial relationships that could be construed as a potential conflict of interest.

## References

[B1] Barsalou L. W. (1999). Perceptual symbol systems. *Behav. Brain Sci.* 22 577–660. 10.1017/S0140525X99532147 11301525

[B2] Cohen Kadosh R. Lammertyn J. Izard V. (2008). Are numbers special? An overview of chronometric, neuroimaging, developmental and comparative studies of magnitude representation. *Prog. Neurobiol.* 84 132–147. 10.1016/j.pneurobio.2007.11.001 18155348

[B3] Dehaene S. Bossini S. Giraux P. (1993). The mental representation of parity and number magnitude. *J. Exp. Psychol.* 122 371–396. 10.1037/0096-3445.122.3.371

[B4] Fias W. Brysbaert M. Geypens F. d’Ydewalle G. (1996). The importance of magnitude information in numerical processing: evidence from the SNARC effect. *Math. Cogn.* 2 95–110. 10.1080/135467996387552

[B5] Fias W. Fischer M. H. (2004). “Spatial representation of numbers,” in *Handbook of Mathematical Cognition* ed. Campbell J. I. (New York, NY: Psychology Press) 43–54.

[B6] Fias W. Lauwereyns J. Lammertyn J. (2001). Irrelevant digits affect feature-based attention depending on the overlap of neural circuits. *Cogn. Brain Res.* 12 415–423. 10.1016/S0926-6410(01)00078-7 11689301

[B7] Fischer M. H. Castel A. D. Dodd M. D. Pratt J. (2003). Perceiving numbers causes spatial shifts of attention. *Nat. Neurosci.* 6 555–556. 10.1038/nn1066 12754517

[B8] Gevers W. Reynvoet B. Fias W. (2003). The mental representation of ordinal sequences is spatially organized. *Cognition* 87 B87–B95. 10.1016/S0010-0277(02)00234-2 12684205

[B9] Gevers W. Reynvoet B. Fias W. (2004). The mental representation of ordinal sequences is spatially organized: evidence from days of the week. *Cortex* 40 171–172. 10.1016/S0010-9452(08)70938-9 15174454

[B10] Gevers W. Verguts T. Reynvoet B. Caessens B. Fias W. (2006). Numbers and space: a computational model of the SNARC effect. *J. Exp. Psychol.* 32 32–44. 10.1037/0096-1523.32.1.32 16478324

[B11] Huber S. Klein E. Moeller K. Willmes K. (2016). Spatial-numerical and ordinal positional associations coexist in parallel. *Front. Psychol.* 7:438 10.3389/fpsyg.2016.00438 PMC4811880 27064216

[B12] Lindemann O. Abolafia J. M. Pratt J. Bekkering H. (2008). Coding strategies in number space: memory requirements influence spatial–numerical associations. *Q. J. Exp. Psychol.* 61 515–524. 10.1080/17470210701728677 18300183

[B13] Loetscher T. Schwarz U. Schubiger M. Brugger P. (2008). Head turns bias the brain’s internal random generator. *Curr. Biol.* 18 R60–R62. 10.1016/j.cub.2007.11.015 18211838

[B14] Moyer R. S. Landauer T. K. (1967). Time required for judgements of numerical inequality. *Nature* 215 1519–1520. 10.1038/2151519a0 6052760

[B15] Pinhas M. Tzelgov J. Ganor-Stern D. (2012). Estimating linear effects in ANOVA designs: the easy way. *Behav. Res. Methods* 44 788–794. 10.3758/s13428-011-0172-y 22101656

[B16] Previtali P. de Hevia M. D. Girelli L. (2010). Placing order in space: the SNARC effect in serial learning. *Exp. Brain Res.* 201 599–605. 10.1007/s00221-009-2063-3 19888566

[B17] Restle F. (1970). Speed of adding and comparing numbers. *J. Exp. Psychol.* 83 274–278. 10.1186/1471-2334-11-269

[B18] Tzelgov J. Zohar-Shai B. Nuerk H.-C. (2013). On defining quantifying and measuring the SNARC effect. *Front. Psychol.* 4:302 10.3389/fpsyg.2013.00302 PMC3667495 23750147

[B19] van Dijck J.-P. Fias W. (2011). A working memory account for spatial–numerical associations. *Cognition* 119 114–119. 10.1016/j.cognition.2010.12.013 21262509

[B20] Van Opstal F. Fias W. Peigneux P. Verguts T. (2009). The neural representation of extensively trained ordered sequences. *Neuroimage* 47 367–375. 10.1016/j.neuroimage.2009.04.035 19376245

[B21] Vicario C. M. (2012). Perceiving number affects the internal random movements generator. *Sci. World J.* 2012 1–6. 10.1100/2012/347068 PMC3353301 22629133

[B22] Walsh V. (2003). A theory of magnitude: common cortical metrics of time, space and quantity. *Trends Cogn. Sci.* 7 483–488. 10.1016/j.tics.2003.09.002 14585444

